# Human semen contains exosomes with potent anti-HIV-1 activity

**DOI:** 10.1186/s12977-014-0102-z

**Published:** 2014-11-19

**Authors:** Marisa N Madison, Richard J Roller, Chioma M Okeoma

**Affiliations:** Department of Microbiology, Carver College of Medicine, University of Iowa, 51 Newton Road, Iowa City, IA 52242-1109 USA; Interdisciplinary Program in Molecular and Cellular Biology, University of Iowa, Iowa City, IA 52242 USA

**Keywords:** Exosome, Semen, HIV-1, mAIDS, HSV

## Abstract

**Background:**

Exosomes are membranous nanovesicles secreted into the extracellular milieu by diverse cell types. Exosomes facilitate intercellular communication, modulate cellular pheno/genotype, and regulate microbial pathogenesis. Although human semen contains exosomes, their role in regulating infection with viruses that are sexually transmitted remains unknown. In this study, we used semen exosomes purified from healthy human donors to evaluate the role of exosomes on the infectivity of different strains of HIV-1 in a variety of cell lines.

**Results:**

We show that human semen contains a heterologous population of exosomes, enriched in mRNA encoding tetraspanin exosomal markers and various antiviral factors. Semen exosomes are internalized by recipient cells and upon internalization, inhibit replication of a broad array of HIV-1 strains. Remarkably, the anti-HIV-1 activity of semen exosomes is specific to retroviruses because semen exosomes blocked replication of the murine AIDS (mAIDS) virus complex (LP-BM5). However, exosomes from blood had no effect on HIV-1 or LP-BM5 replication. Additionally, semen and blood exosomes had no effect on replication of herpes simplex virus; types 1 and 2 (HSV1 and HSV2). Mechanistic studies indicate that semen exosomes exert a post-entry block on HIV-1 replication by orchestrating deleterious effects on particle-associated reverse transcriptase activity and infectivity.

**Conclusions:**

These illuminating findings i) improve our knowledge of the cargo of semen exosomes, ii) reveal that semen exosomes possess anti-retroviral activity, and iii) suggest that semen exosome-mediated inhibition of HIV-1 replication may provide novel opportunities for the development of new therapeutics for HIV-1.

## Background

Exosomes are endogenous carriers of genetic cargo [1,2] and they can transfer their cargo to recipient target cells [3]. Exosomes are released by a variety of cell types and have been isolated from diverse biological fluids, such as blood plasma [4], urine [5], saliva [6], breast milk [7,8], bronchoalveolar lavage fluid [8], and seminal plasma [9]. Human semen contains a heterogenous population of membranous nanovesicles produced by tissues of the male genital tract (MGT) [10]. Collectively, the diverse array of semen-derived nanovesicles is most often termed prostasomes. Owing to the fact that these nanovesicles arise from diverse cell types within the MGT, it is more appropriate to refer to them as nanovesicles or exosomes. Due to their endosomal origin, exosomes are endowed with a repertoire of host proteins, including tetraspannins (CD9, CD63, and CD81) [11,12], miRNAs, and mRNAs [13]. The RNA “cargo” of exosomes may be significantly different from that of the originating parental cell content [14-16].

Exosomes from different origins are internalized by cells [17] where they may mediate intercellular communication, immunoregulatory effects, and regulation of microbial pathogenesis [18,19]. The ability of liver nonparenchymal cell derived exosomes to modulate host response against viral infection was previously suggested for hepatitis B virus-infected hepatocytes [19]. Semen-derived nanovesicles, hereafter referred to as semen exosomes (SE), have been implicated in varied processes relating to spermatozoa function and protection in the vaginal milieu [20], but their role in viral infectivity has not previously been demonstrated. It is thus conceivable that the role of exosomes could be deduced from their origin. Therefore, SE may function to either enhance or block replication of sexually transmitted pathogens, such as HIV-1.

HIV-1 transmission worldwide primarily occurs through sexual contact and semen is the primary vector [21]. However, sexual transmission of HIV-1 requires a high number of exposures [22,23]. It is known that post-coitus, exposure of the female reproductive tract (FRT) to semen results in immunomodulatory events that influence the outcome of HIV-1 replication within the genital mucosa, indicating that semen contains factors that may enhance or abate HIV-1 infection [24-27].

Here we study the role of SE in HIV-1 replication. We show that SE is internalized by recipient cells and upon internalization SE blocks HIV-1 replication. SE-mediated inhibition of virus replication occurred post-entry and involves impairment of viral RNA reverse transcription. Thus, our data identify exosomes from semen as a critical factor that may reduce efficacy of HIV-1 transmission and highlight a unique opportunity for additional studies on the cargo composition and function of semen exosomes.

## Results

### Human semen contains exosomes loaded with proteins and functional mRNA

**Figure 1:**
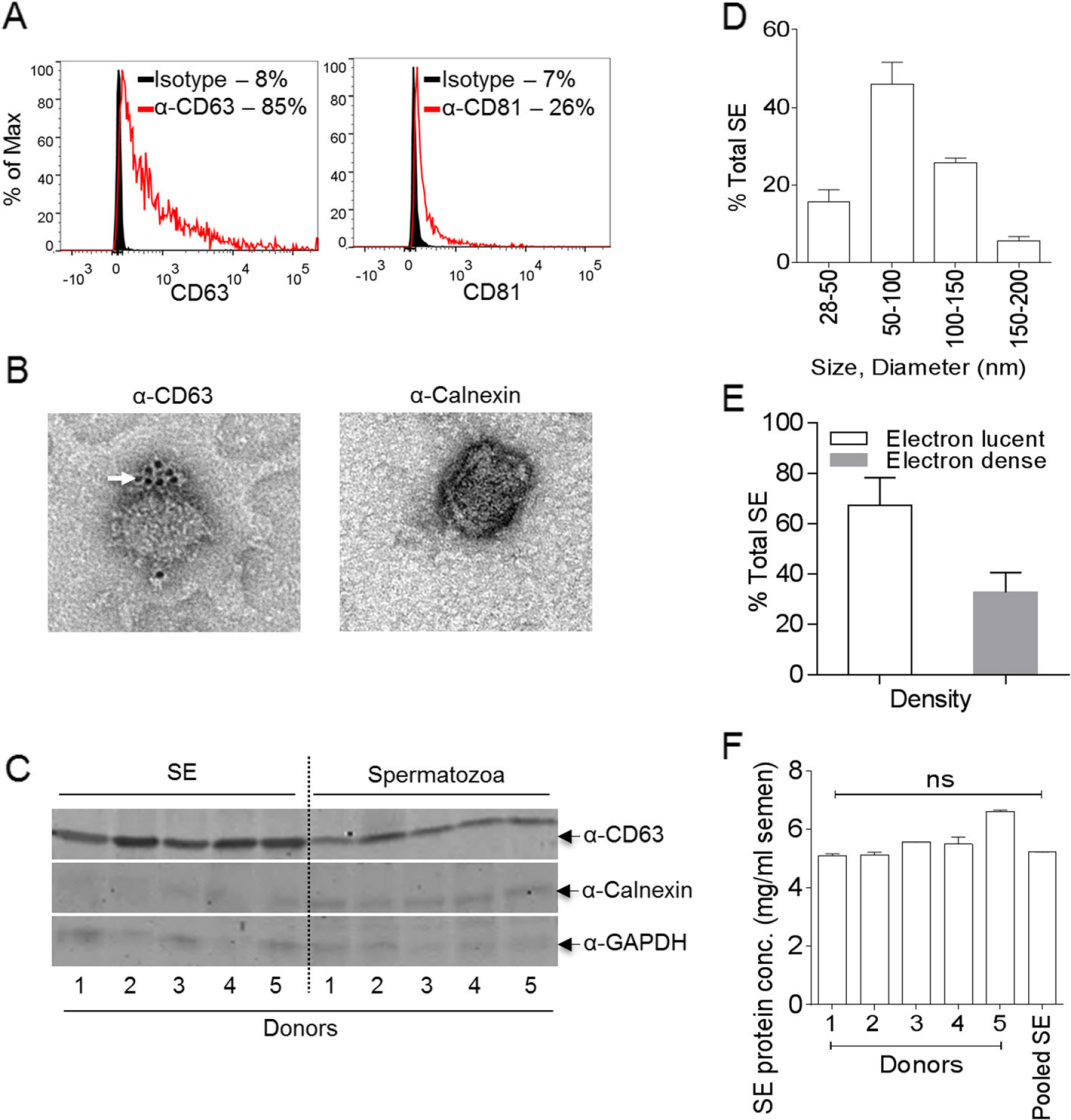
**Characteristics of human semen exosomes. (A)** Fluorescence-activated cell sorting (FACS) analysis of pooled (n =20) SE incubated with α-MHC-II antibody coated latex beads (4 μm) and stained for CD63 (Left, red histogram) and CD81 (Right, red histogram). Isotype control antibody is represented by black histograms. **(B)** Left, transmission electron microscopy (TEM) of pooled (n =20) semen exosome (SE) stained for CD63 (α-CD63) and detected by 6 nm gold-labeled secondary antibody (arrow). Right, TEM of pooled (n =20) SE stained for Calnexin (α- Calnexin) and counterstained with 10 nm gold-labeled secondary antibody. Calnexin was not detected. **(C)** Western blot of SE (n =5) and paired spermatozoa protein extracts, stained for CD63, Calnexin, and GAPDH. Pooled SE population (n =89) were categorized based on **(D)** size and **(E)** electron density. **(F)** SE protein concentrations from different donors range between 5.3 – 6.8 mg/ml of semen.

**Figure 2:**
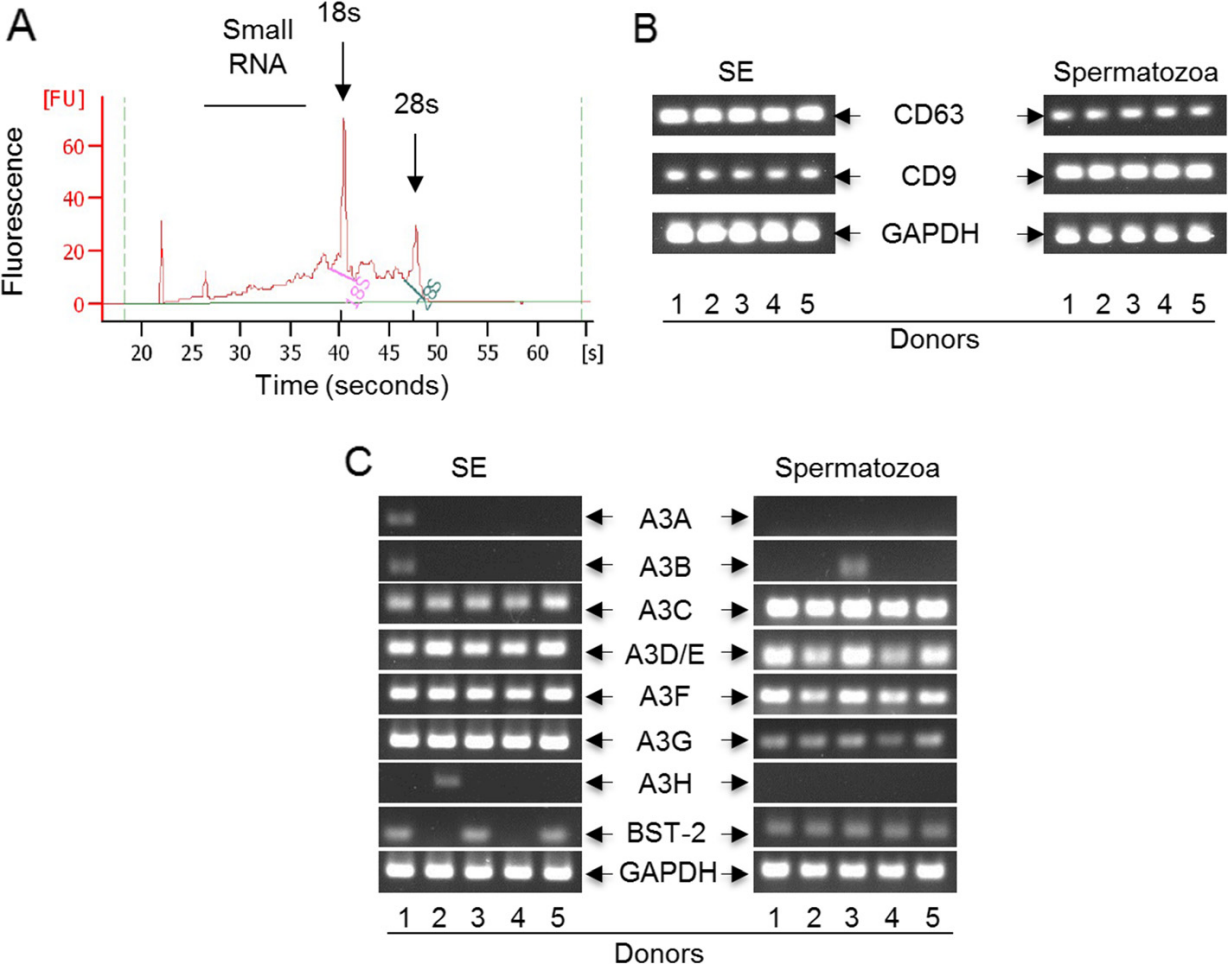
**Human semen contains exosomes loaded with coding mRNA. (A)** RNA analysis with the Bioanalyzer revealed that SE contains different RNA species. Arrows show peaks for 18 s, 28 s, and the horizontal line represents small RNA. **(B** and **C)** PCR analysis of tetraspanin (CD9 and CD63) mRNA and host restriction factors (HRFs) mRNA present in paired SE and spermatozoa samples. Numbers 1 to 5 represent different donors. GAPDH is present in SE and spermatozoa and was used as loading control.

FACS, TEM, and immunoblot analysis revealed that SE is enriched in tetraspanin proteins CD63 and CD81, but devoid of contaminating endoplasmic reticulum-derived marker calnexin (Figure 1A, B and C), suggesting that we have pure populations of exosomes. Structurally, we found that SE populations are morphologically distinct with respect to size [28] and electron density [29] (Figure 1D and E). The protein concentrations within SE are similar amongst donors and range between 5.3 – 6.8 mg/ml of semen (Figure 1F). Additional characterization reveals that SE contains diverse RNA species, including small RNA and mRNA (Figure 2A), supporting a previous report [30]. The ability to synthesize complimentary RNA capable of sustaining PCR amplification of tetraspanins (Figure 2B) and various antiviral factors, such as host restriction factors (HRFs); that block replication of diverse retroviruses (Figure 2C) demonstrates that SE contains polyadenylated RNA capable of supporting gene expression. The presence of HRF mRNA, including Apobec3 (A3) and BST-2 in SE is intriguing since exosome-mediated transfer of RNA from donor to recipient cells results in functional RNA-related biological effects in the recipient cells [15,31].

### Semen exosomes inhibit HIV-1 replication

**Figure 3:**
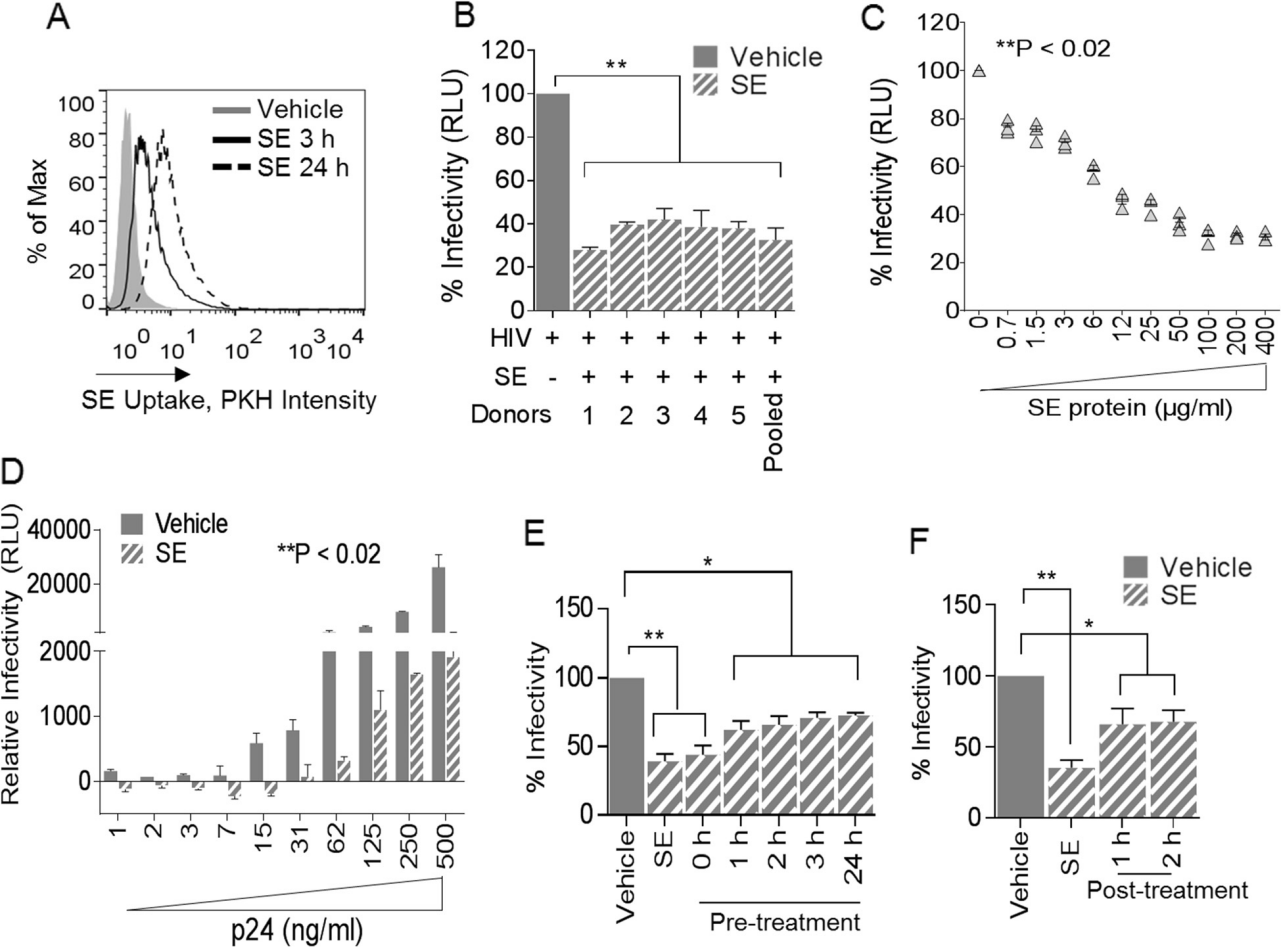
**Semen exosomes inhibit HIV-1 replication. (A)** TZM-bl cells were evaluated for cellular uptake of green fluorochrome-labelled SE by FACS. PKH67Green-labelled PBS (gray histogram) was used as control. **(B)** TZM-bl indicator cells challenged with HIV-1_NL4.3_ pre-incubated with PBS Vehicle or with 100 μg/ml individual donor SE, or pooled (n =20) SE. Infectivity was determined by luminescence output with control set at 100% infection. **(C)** TZM-bl indicator cells challenged with HIV-1_NL4.3_ pre-incubated with increasing concentrations (0–400 μg/ml) of SE and analyzed for infectivity by luminescence output. **(D)** TZM-bl cells were challenged with 100 μg/ml of SE pre-incubated with increasing concentrations (1 – 500 ng/ml p24) of HIV-1_NL4.3_. Infectivity was determined by luminescence output. **(E)** HIV-1_NL4.3_ was pre-incubated for 1 h with vehicle (gray bar, vehicle) or with SE (SE), or added simultaneously with SE (0 h), or added prior (1 h, 2 h, 3 h, and 24 h) to HIV-1 challenge. Infectivity on TZM-bl cells was measured by luminescence emission with vehicle set at 100% infection. **(F)** HIV-1_NL4.3_ was pre-incubated for 1 h with vehicle (gray bar, vehicle) or with SE (SE), or added directly to TZM-bl indicator cells at 1 h or 2 h post-infection. Infectivity was measured by luminescence emission with vehicle set at 100%. Student’s t test was used for all samples, significance was taken at *P <0.05, **P <0.02. Experiments were repeated at least 3 times with similar results.

To assess the role of SE in HIV-1 replication, we first evaluated whether SE is internalized by target cells. Uptake of PKH67Green-labeled SE by HIV-1 target cells, TZM-bl was evident within 3 h of exposure of TZM-bl to SE; with increased uptake observed at 24 h post-exposure (Figure 3A). We further determined that SE has no effect on recipient TZM-bl cell viability because cytotoxicity was not observed upon exposure of cells to SE, even at concentrations as high as 1000 μg/ml (data not shown).

Analysis of HIV-1 Tat-dependent luminescence in the presence of SE showed marked suppression of HIV-1 replication by various donor SE that were pre-incubated with HIV-1 in comparison to control cells exposed to HIV-1 that was pre-incubated with PBS alone (Figure 3B). Pooled donor SE (n =20) with concentrations ranging from 0.7 – 400 μg/ml diminished HIV-1 infectivity in a dose-dependent manner (Figure 3C) and SE (100 μg/ml) potently blocked a wide range of HIV-1 viral inoculum (Figure 3D). While pre-incubating SE with HIV-1 for 1 h at 37°C resulted in the most efficacious inhibition (Figure 3B-D), simultaneous addition of SE and virus to cells, or pre-treatment of cells with SE for various times prior to viral challenge partially protected cells against HIV-1 in a time dependent manner (Figure 3E). In addition, SE conferred partial protection when added to TZM-bl cells at different time points after viral challenge (Figure 3F).

### Semen exosomes inhibit replication of HIV-1 in various cell types

**Figure 4:**
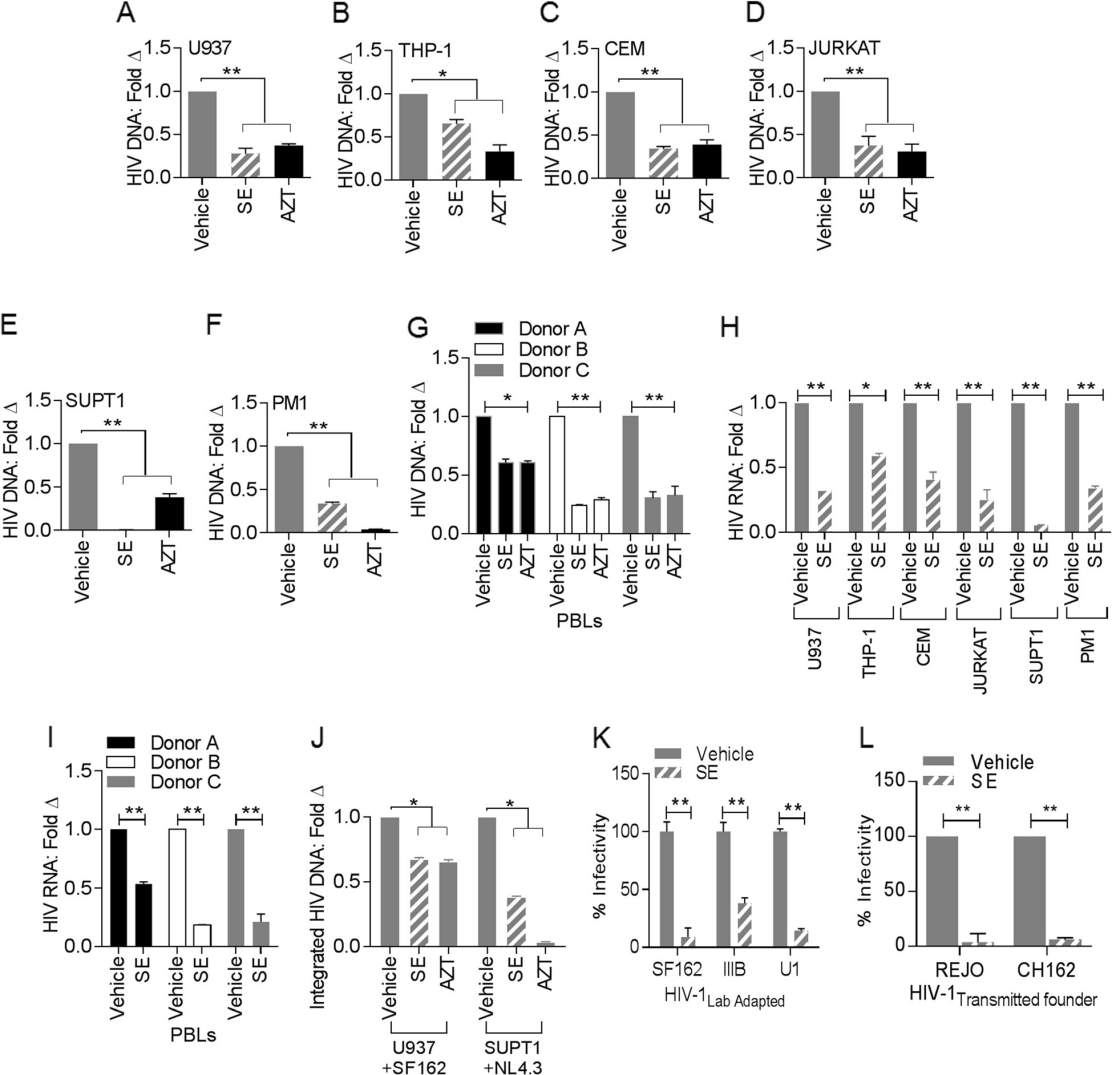
**Cell type- and virus strain- independent restriction of HIV-1 replication by semen exosomes. (A** **and** **B)** Monocytic cell lines **(A)** U937 and **(B)** THP1; **(C** **to** **F)** T lymphocyte cell lines; **(C)** Rev-CEM, **(D)** JURKAT, **(E)** SupT1, **(F)** PM1, and **(G)** PBL (from 3 different healthy donors) pretreated ± AZT for 2 h were exposed to HIV-1_SF-162_ or HIV-1_NL4-3_ pre-incubated with PBS or SE for 1 h. Total DNA isolated from cells was examined for levels of HIV-1 Gag DNA 24 h later by qPCR. **(H)** U937, THP1, Rev-CEM, JURKAT, SupT1, and PM1 were exposed to HIV-1_SF-162_ or HIV-1_NL4-3_ pre-incubated with PBS or SE for 1 h. Total RNA was examined for levels of HIV-1 Gag RNA 24 h later by RT-qPCR. **(I)** PBL from 3 healthy donors were pretreated ± AZT for 2 h and exposed to HIV-1_NL4-3_ pre-incubated with PBS or SE. Infectivity was assessed by qPCR and RT-qPCR for detection of HIV-1 Gag DNA or RNA respectively. **(J)** Total DNA was examined for integrated HIV-1 DNA in U937 cells exposed to HIV-1_SF-162_ or SupT1 cells exposed to HIV-1_NL4-3_ in the presence or absence of SE or AZT by Alu-PCR. **(K)** TZM-bl cells exposed to R5-monotropic clade B (SF162) or X4-monotropic clade B (IIIB and U1) pre-incubated with PBS or SE. HIV-1 pre-incubated with PBS was set at 1. **(L)** TZM-bl cells exposed to transmitted/founder (T/F) molecular infectious clones (REJO and CH162) of HIV-1 pre-incubated with PBS or SE were examined for infectivity by luminescence output. HIV-1 pre-incubated with PBS was set at 1. **P < 0.02 and Student's t test was used for all samples. Data are expressed as mean ± SD and presented as fold change of vehicle. Experiments were repeated at least three times with similar results.

To determine whether SE is capable of blocking HIV-1 replication in cells with physiologic relevance to HIV-1 replication, we quantified HIV-1 proviral DNA and viral RNA from different cell types in the presence or absence of SE. This experiment also assessed cell type specific responses to SE-mediated inhibition of HIV-1. Control cells were pre-treated with the reverse transcription inhibitor AZT prior to infection. Levels of HIV-1 DNA (Figure 4A-F) and HIV-1 RNA (Figure 4H) revealed that SE potently diminished HIV-1 infectivity in U937 and THP-1 monocytic cell lines (Figure 4A,B and H) as well as in various T cell lines, including CEM (Figure 4C and H), Jurkat (Figure 4D and H), SupT1 (Figure 4E and H) and PM1 (Figure 4F and H). Importantly, SE significantly restricted HIV-1 replication in primary peripheral blood lymphocytes (PBL) in a donor-dependent manner as shown by significant reduction in levels of HIV-1 DNA (Figure 4G) and HIV-1 RNA (Figure 4I). Furthermore, SE abrogated integration of viral DNA into the host chromosomal DNA (Figure 4J). Indeed, SE inhibited HIV-1 replication and integration in all cell lines queried. However, we observed modest cell type-dependent responses. Of note, SE-mediated inhibition of HIV-1 replication paralleled inhibition imposed by AZT (especially in PBLs [Figure 4G]; and U937 cells [Figure 4A and J]); although some cell type-dependent variability was also observed.

### Lab-adapted and clinical isolates of HIV-1 are susceptible to SE inhibition

Compared to HIV-1 alone, SE significantly compromised replication of lab-adapted and transmitted/founder (T/F) viruses as indicated by HIV-1 Tat-dependent luminescence output (Figure 4K and L). These results signify that the anti-HIV-1 effect of SE extended to different R5- and X4-tropic lab-adapted and transmitted/founder (T/F) viruses regardless of tropism.

### Inhibition of retrovirus replication is specific to semen exosomes

**Figure 5:**
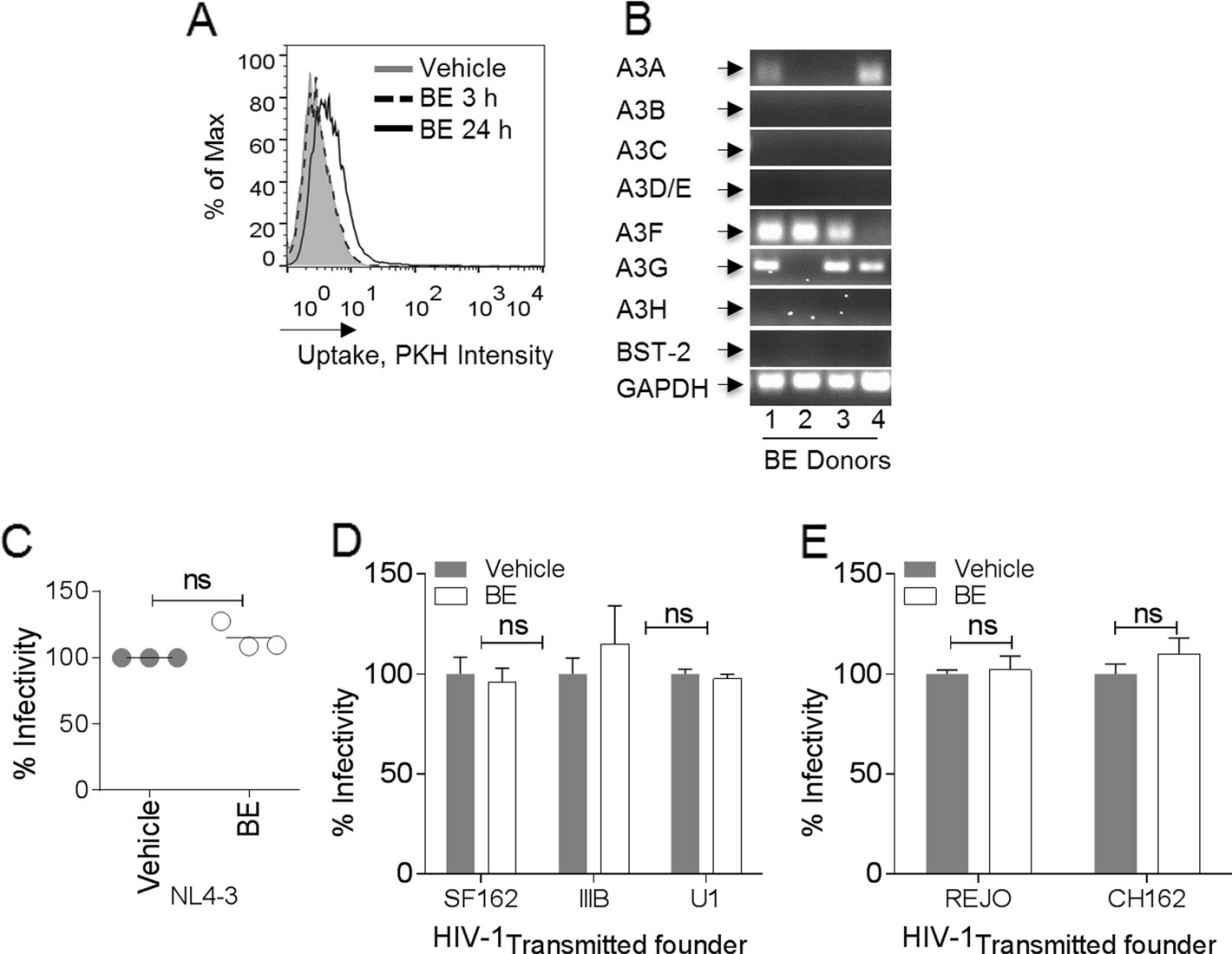
**HIV-1 is not sensitive to blood exosomes. (A)** TZM-bl cells were evaluated for cellular uptake of green fluorochrome-labelled blood serum exosome (BE) by FACS. PKH67Green-labelled PBS (gray histogram) was used as control. **(B)** PCR analysis of host restriction factor (HRF) mRNA present in BE donor samples. Numbers 1 to 4 represent different donors. GAPDH is present in BE and was used as loading control. **(C)** TZM-bl indicator cells challenged with HIV-1_NL4-3_ pre-incubated with PBS or with 100 μg/ml donor (n =3) BE. Infectivity was determined by luminescence output with control set at 100% infection. **(D)** TZM-bl cells exposed to R5- and X4-tropic lab-adapted HIV-1 isolates, including R5-monotropic clade B (SF162), X4-monotropic clade B (IIIB and U1) pre-incubated with PBS or BE. Infectivity was assessed by luminescence expression. Vehicle infectivity was set at 100%. **(E)** TZM-bl cells exposed to transmitted founder (T/F) molecular infectious clones (REJO and CH162) of HIV-1 pre-incubated with PBS or BE were examined for infectivity by luminescence output. Differences between treatments were not significant (ns). Experiments were repeated at least three times with similar results.

SE is internalized by target TZM-bl cells (Figure 3A) and we show that exosomes from blood serum (BE) are also internalized (Figure 5A). Unlike SE that was internalized as early as 3 h post addition to cells, BE was not internalized by TZM-bl cells at 3 h. However, BE internalization was evident 24 h later (Figure 5A). Like SE, BE also contains polyadenylated RNA capable of supporting gene expression as evidenced by the presence of HRF transcripts (Figure 5B). It is worth mentioning that similar to SE (Figure 2C), there is donor-dependent variability in BE content of HRFs (Figure 5B). To determine whether SE-mediated block on HIV-1 replication can be achieved with BE, TZM-bl cells were exposed to HIV-1_NL4.3_ that had been pre-incubated with vehicle or BE. In comparison to SE (Figure 4), BE-treated viruses retained infectivity (Figure 5C). Level of infectivity observed in the presence of BE paralleled that seen with HIV-1 alone (Figure 5C), corroborating a previous report [32]. A similar trend was observed when BE was pre-incubated with various lab-adapted (Figure 5C-D) and T/F viruses (Figure 5E).

**Figure 6:**
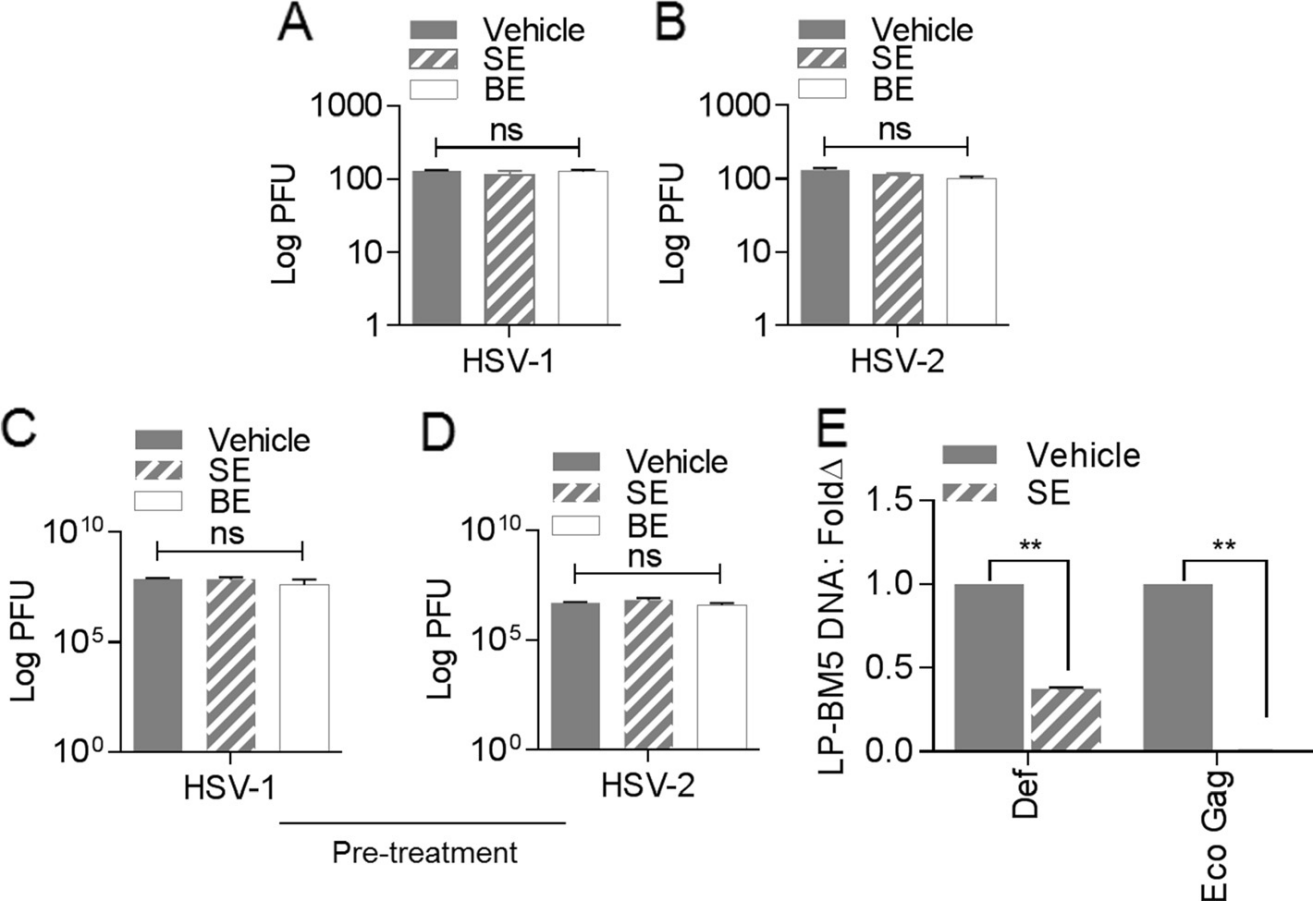
**HSV −1 and HSV −2 are refractory to the antiviral activity of semen exosomes. (A** **and** **B)** Infectivity of HSV-1 or HSV-2 on HEp-2 cells was determined by counting plaque forming units (PFU) two days post infection with 2 X 10^5^ PFU of HSV-1 or HSV-2 pre incubated for 1 h at 37°C with PBS vehicle (gray bar), 100 μg/ml semen exosomes or 100 μg/ml blood exosomes (white bar). Virus titre is shown as particle forming unit (PFU) per ml. **(C** **and** **D)** HEp-2 cells were preincubated at 37°C for 1 h with PBS vehicle (gray bar), 100 μg/ml SE or 100 μg/ml BE (white bar) followed by challenge with HSV-1 or HSV-2 (MOI =5) at 37°C for 1 h. Viral replication was allowed to ensue for 18 h. Progeny virus was collected and titered on Vero cells for determination of virus titre by plaque assay. Data are mean ± SD and are reported as plaque forming units (PFU). Student’s t test was used for all samples; significance was taken at P ≤0.05. ns = not significant. Experiments were repeated at least 3 times with similar results. **(E)** Murine splenocytes are susceptible to SE-mediated inhibition of LP-BM5 replication *ex vivo*: Naïve splenocytes were infected with LP-BM5 pre-incubated with vehicle or SE. 96 h post challenge, total DNA was isolated and used to evaluate viral load by quantitative PCR detection of proviral DNA. Results were normalized for GAPDH. Data are mean ± SD expressed as fold change of vehicle set at 1. *P <0.02, Student’s t test was used for all samples. Experiments were repeated at least three times with similar results.

Since SE but not BE blocked HIV-1 replication, we assessed whether SE and/or BE could inhibit replication of viruses other than HIV-1. We pre-incubated SE or BE with herpes simplex viruses (HSV) -1 and −2 which are common sexually transmitted viruses. Analysis of HSV replication by plaque assay on HEp-2 cells reveals that SE and BE had no effect on HSV replication (Figure 6A and B). Additionally, pretreatment of HEp-2 cells with SE or BE prior to infection had no effect on herpesvirus plaque formation (Figure 6C and D). These data indicate that exosomes from either semen or blood are unable to inhibit HSV replication in cells.

To examine whether SE blocks replication of another sexually transmitted retrovirus [33], we pre-incubated SE with the LP-BM5 MLV murine AIDS virus complex or PBS and subsequently challenged naïve murine splenocytes with LP-BM5 pre-incubated with SE or PBS. Analysis of LP-BM5 MLV replication by RT-qPCR reveals that SE suppressed LP-BM5 proviral DNA compared to the vehicle control (Figure 6E). These data indicate that the anti-viral effect of SE is perhaps an intrinsic feature of SE on sexually transmitted retroviruses but not herpesviruses.

### Semen exosomes do not impair the concentration of HIV-1 that enters target cells

**Figure 7:**
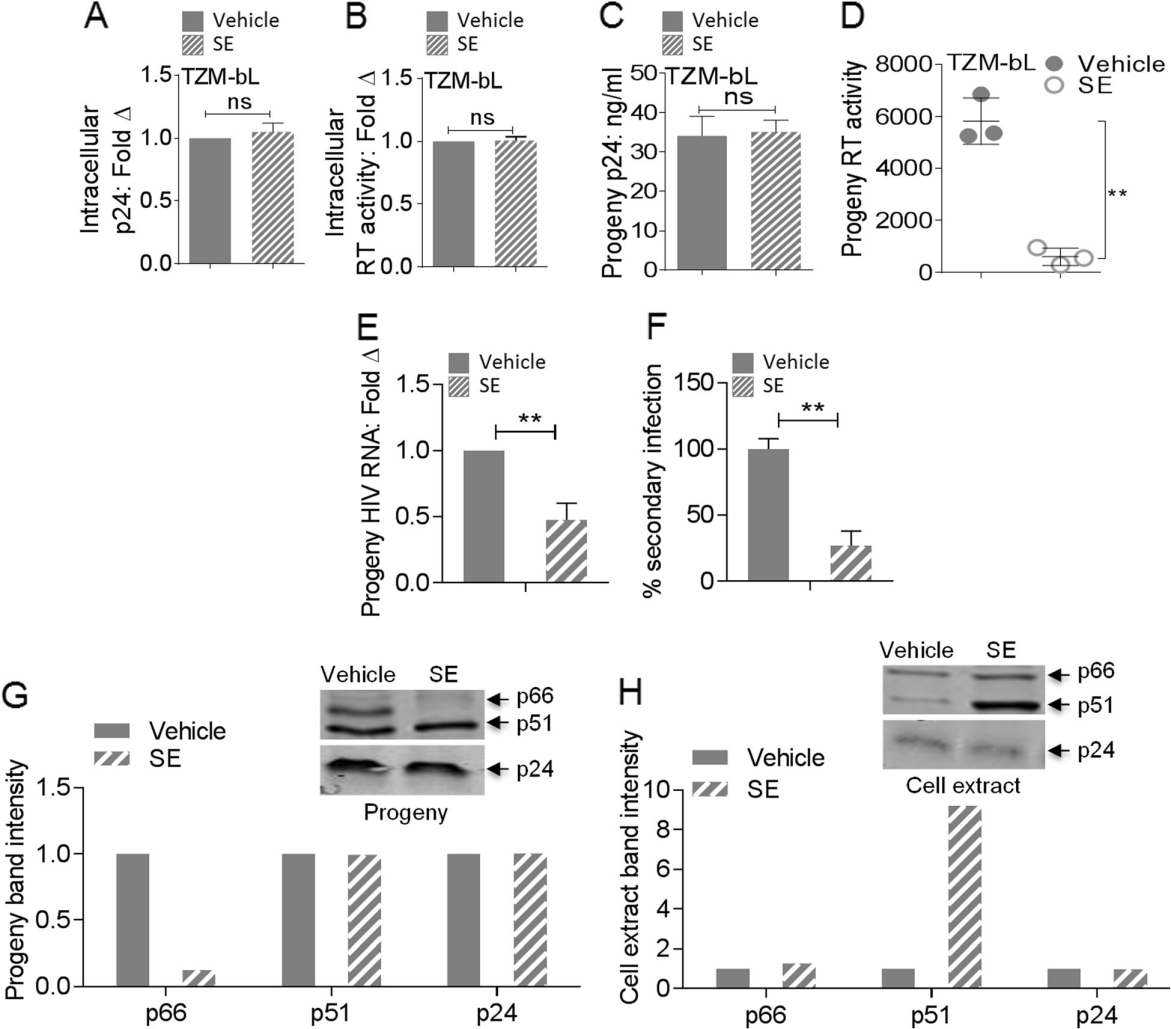
**Post-entry effect of semen exosomes on HIV infectivity.** Intracellular HIV-1NL4.3 **(A)** p24 ELISA and **(B)** RT activity in TZM-bl cells 3 h post-challenge. Data ± SD are plotted as fold change of vehicle set at 1. **(C)** Cell free supernatant progeny of HIV-1_NL4-3_ produced from TZM-bl 24 h post-challenge depicting levels of p24 Gag as assessed by p24 ELISA. **(D)** Intravirion RT activity of HIV-1 progeny produced from TZM-bl 24 h post-infection. Data ± SD are expressed as fold change of vehicle set at 1 for all graphs. **(E)** Intravirion Gag RNA expression in cell-free supernatant progeny of HIV-1_NL4-3_ produced from TZM-bl reporter cells 24 h post-challenge with HIV-1 pre-incubated with vehicle or SE. Total progeny viral RNA was isolated from cell-free supernatant, converted to cDNA and examined for HIV-1 Gag expression by RT-qPCR. Results were normalized for GAPDH. Data ± SD are expressed as fold change of vehicle set at 1. **(F)** Secondary infectivity of HIV-1 viral progeny isolated from cell free supernatant of TZM-bl reporter cells 24 h post-challenge with HIV-1 pre incubated with vehicle or SE. Input was normalized for p24, added to fresh TZM-bl reporter cells and infectivity was evaluated by luminescence output 24 h post-challenge. Data ± SD are shown as % secondary infectivity with progeny generated in the presence of vehicle set at 100%. For panels **A-F**, ns = not significant, **P < 0.02, Student's t test was used for all samples. **(G** **and** **H)** Immunoblot using 30 μg of viral protein (progeny) or cellular protein from TZM-bl cells challenged for 24 h with HIV-1_NL4-3_ ±SE and probed with antibody against HIV-1 RT or p24 Gag to detect **(G)** intravirion or **(H)** intracellular HIV-1 RT and p24 Gag proteins.

To identify the mechanism of retroviral suppression by SE, we used HIV-1 infection of TZM-bl target cells to assess whether SE impairs the concentration of HIV-1 that enters cells. For this purpose, TZM-bl exposed to HIV-1 in the presence or absence of SE for 3 h were washed and trypsinized [34] to remove input virus prior to cell lysis and analysis of intracellular HIV-1 p24 concentration using p24 antigen ELISA. We found that equivalent concentrations of HIV-1 entered cells in the presence and absence of SE (Figure 7A). In parallel, reverse transcriptase (RT) activity in these cells at the 3 h time point suggested that SE did not compromise viral fitness post entry as measured by RT activity (Figure 7B). These results are consistent with SE-mediated post-entry block on HIV-1 infectivity.

### Semen exosomes compromise intravirion reverse transcriptase activity

Because SE had no effect on the concentration of virus that entered target cells at 3 h post infection (Figure 7A and B) and SE compromised proviral DNA integration and gene expression at 24 h post infection (Figures 4), we assessed the effect of SE on the concentration and fitness of progeny virions generated 24 h following infection. Surprisingly, the concentration of progeny generated in the presence and absence of SE were consistently equivalent at 24 h post infection as determined by p24 ELISA (Figure 7C). However, intravirion RT activity, a measure of viral fitness, was reduced in progeny produced at 24 h post infection in the presence of SE (Figure 7D). Additionally, the effect of SE on HIV-1 RNA incorporated into nascent progeny virions 24 h post infection was examined by qPCR of Gag RNA expression. Progeny generated in the presence of SE contained ≤50% Gag RNA compared to vehicle control (Figure 7E).

Because both progeny RT and RNA were impaired by SE, we assessed progeny fitness by infecting TZM-bl reporter cells with p24 normalized progeny generated from 24 h infection with HIV-1 ± SE. Progeny generated in the presence of SE demonstrated a greater than 50% reduction in secondary infectivity, revealing that SE diminished replication of HIV-1 by compromising the fitness of progeny virions (Figure 7F).

### Semen exosomes alter HIV-1 RT protein composition

To further probe the effect of SE on HIV-1 RT, we evaluated intravirion RT protein composition by Western blotting. We found differences in the ratios of RT heterodimer (p51 and p66) but not p24 within progeny virions generated 24 h post infection in the presence and absence of SE.

Further analysis revealed that when normalized to p24 and compared to vehicle control, SE decreased intravirion p66 protein and had no effect on p51 protein (Figure 7G; blot and bars―compare the solid and stripped bars). Intracellularly, SE increased p51 and had no effect on p66 (Figure 7H; blot and bars, compare the solid and stripped bars). Interestingly, progeny virion and cellular p24 Gag levels were equivalent in the presence and absence of SE both in the progeny and cell extract; an indication that i) SE may not have affected Gag incorporation and processing (Figure 7G and H, and bars); and ii) same concentration of HIV-1 is generated in the presence or absence of SE.

## Discussion

Exosomes deliver their cargo to recipient cells and subsequently modulate host cell function. The mechanisms involved in exosomal modulation of various intracellular events are only beginning to be understood but clearly involve internalization and cargo delivery [14-19]. Indeed, exosomes from different sources possess antiviral activities. This is evident in the case of exosomal delivery of antiviral IFNα to HBV infected hepatocytes [19] and blockade of HIV-1 infection of dendritic cells by breast milk exosomes [32]. The present study aimed to provide answers as to whether exosomes from semen (i) are internalized by target cells and (ii) possess antiviral activity against viruses transmitted via semen.

We found that human semen exosomes (SE) are efficiently internalized by target cells as early as 3 hours after exposure of cells to SE. Importantly, SE restricted replication of a wide range of HIV-1 strains and the murine AIDS virus complex LP-BM5. Inhibition of retrovirus replication by SE is specific as exosomes from blood (BE) had no effect on these viruses. Moreover, SE did not restrict replication of HSV types −1 and −2, demonstrating that the SE antiviral effect is specific to retroviruses. The anti-HIV-1 effect of SE appeared to occur after virus entry.

Although equivalent amounts of HIV-1 (as demonstrated by HIV-1 p24 Gag levels) entered host cells in the presence and absence of SE at 3 h post infection, later in the infection process p24 Gag levels remained unchanged while viral RT activity was drastically reduced in HIV-1 progeny generated in the presence of SE. Unlike p24, which measures total virus production, including infectious, noninfectious, and decaying viral particles and only requires the presence of an intact epitope in the capsid/p55 Gag, RT is the best surrogate measure of infectious virus as it measures a specific enzyme function [35]. Therefore, our data suggests that SE impairs HIV-1 DNA and RNA expression and progeny infectivity but has no effect on the amount of viral progeny produced from infected cells. Although it is yet to be determined how and why Gag protein level remained unchanged while RNA level is diminished by SE, it is worth noting that others have observed similar trends in viral nucleic acid and cognate protein expression. It was previously demonstrated that HIV-1 Gag protein expression can be rescued while Gag mRNA expression is depleted [36], and that steady state Gag protein is sufficient to be incorporated into progeny virions in the absence of *de novo* Gag synthesis [37].

The molecular mechanism for the antiretroviral effect of SE on HIV-1 replication is not fully understood but appears to involve alteration in intravirion RT activity and protein composition. The HIV-1 RT is a heterodimer composed of p66 and p51 kDa polypeptides. Appropriate association of these subunits is required for RT enzymatic activity because monomeric subunits lack polymerase activity [38,39]. In our study, p66 RT subunit was absent from virions generated in the presence of SE. Additionally, SE-generated progeny viruses contain less Gag RNA but p24 did not change.

The observation that HRFs mRNAs, such as Apobec3 are part of SE cargo is intriguing, as Apobec3 is a potent antiretroviral [33,40-44]. Apobec3 mediates inhibition of retroviruses by deamination-dependent and independent mechanisms, the later involving inhibition of RT by Apobec3 [43,45-47]. Consistent with this idea, our data show that SE drastically decreased HIV-1 RT activity. While a previous report showed that exosomal Apobec3G released by H9 T lymphocyte cells blocked HIV-1 infection [18], the role of Apobec3 or other HRFs mRNA enwrapped in SE is yet to be determined.

Follow up studies are required to gain understanding of the exact mechanism of SE-mediated HIV-1 inhibition. It is also important to determine whether, like SE from healthy donors, SE from HIV-1 positive individuals contains antiviral mRNA cargo and suppresses HIV-1 infectivity.

## Conclusions

Our findings suggest that a major function of SE is to counteract HIV-1 replication by impairing viral infectivity through the generation of progeny viruses with defect in RT. The fact that HIV-1 is susceptible to antiviral effects of SE suggests a point of vulnerability for the virus that could be therapeutically exploited. Thus, this study serves as a paradigm for understanding the functions of exosomes secreted into human semen.

## Methods

### Ethics statement

This study involves the use of existing human specimens (semen, serum, and PBLs) and therefore is not human subjects’ research. Barthelemy Konan and Dr. Melanie Freeman of the Reproductive Specialty Laboratory of Middle Tennessee, Nashville provided de-identified samples of human semen. These samples were discarded from routine examinations and not linked to any identifiers. Healthy HIV-1-negative subjects were invited to participate in the study. Following written informed consent, blood was obtained for exosome and PBL isolations. This study was approved by the University of Iowa Institutional Review Board.

Experiments involving the use of mice were performed in accordance to NIH guidelines, the Animal Welfare Act, and US federal law. Experiments were approved by the University of Iowa Animal Care and Use Committee (IACUC). All mice were housed according to the policies of the Institutional Animal Care and Use Committee of the University of Iowa.

### Acquisition of human semen

Semen samples were collected by dry manual stimulation and ejaculation into sterile polypropylene tubes. Samples were stored at room temperature for 30 min to promote liquefaction and then centrifuged for 10 min at 1000 × *g* at 4°C to pellet spermatozoa. Spermatozoa-containing pellets were either discarded or used as paired samples. Where indicated, seminal plasma samples were pooled. Seminal plasma samples were stored at -80°C until required for exosome purification.

### Exosome purification

#### ExoQuick

Seminal plasma samples were thawed and centrifuged at 2000 × *g* for 15 min followed by centrifugation at 10,000 × *g* for 30 min to pellet cellular debris. ExoQuick (SBI) exosome precipitation reagent was used according to the manufacturer’s instructions. Briefly, ExoQuick was added at a ratio of 4:1 (seminal plasma/ExoQuick) followed by resuspension by inversion and incubation at 4°C overnight. The seminal plasma/ExoQuick mixture was centrifuged at 1500 × g for 30 minutes. Supernatant was removed and the exosome pellet in ExoQuick was washed in PBS three times with centrifugation at 1500 × g for 15 minutes, diluted 50% in PBS, aliquoted, quantified by the Bradford method and stored at -80°C until use. Exosomes from blood serum were also purified with ExoQuick. While the polymer-based ExoQuick precipitation solution is gaining popularity as a technically facile and relatively less time consuming method for exosome purification [48-50], we also performed our experimentation with exosomes purified via more conventional sucrose gradient differential ultracentrifugation methods as previously described [18,51].

#### Sucrose gradient differential ultracentrifugation

Briefly, clarified seminal plasma was diluted 50% in PBS, 0.45 μm filtered and ultracentrifuged at 100,000 × *g* to pellet exosomes using a SW41Ti rotor. Exosome pellets were washed by resuspension in PBS and ultracentrifugation at 100,000 × *g* for 1 h. Pellets were resuspended in PBS, mixed with 2.5 M/L sucrose, placed on the bottom of SW41Ti ultracentrifuge tubes and overlaid with 2 M/L followed by 0.25 M/L sucrose. After 16 h of ultracentrifugation at 100,000 × *g* (SW41Ti rotor, Beckman Coulter), exosomes were collected from the main band at the 2 M/L and 0.25 M/L interface, resuspended in PBS and pelleted by ultracentrifugation for 30 min at 100,000 × *g*. The exosome pellets were resuspended in a volume of sterile PBS equivalent to the original volume of undiluted seminal plasma, aliquoted, protein concentration quantified by the Bradford method (Bio-Rad), and stored at −80°C until use. We determined that the method of purification did not alter the ability of SE to inhibit HIV replication.

### Viruses

HIV-1_pNL4.3_ was obtained from the NIH AIDS Reagent Program and expanded by transfecting HEK 293 cells using Lipofectamine according to the manufacturer’s instructions (Invitrogen). U1 virus stock was produced from U937 cells chronically infected with U1 R5-tropic HIV-1 obtained from the NIH AIDS Reagent Program. The U1/HIV-1 cells were induced for 48 h using 100 ng/ml phorbol 12-myristate 13-acetate (PMA, Sigma-Aldrich). Thereafter, supernatants were collected and centrifuged at 1500 × *g* for 5 min to remove cellular debris then 0.45 μm filtered. HIV-1 was purified from culture supernatants of infected cells by ultracentrifugation at 30,000 rpm for 2 h through a 20% sucrose cushion, as previously described [52]. Viral pellets were resuspended in RPMI and stored at −80°C until use. Transmitted/founder HIV-1 infectious molecular clones, REJO and CH162 [53] in addition to pSF162 plasmid were provided by Dr. Wendy Maury of the University of Iowa and produced by PEI transfection of HEK 293 cells. Culture supernatant was concentrated by ultracentrifugation over a sucrose cushion as described above. All purified and pelleted viral stocks were treated with DNAse, resuspended in RPMI with 10% exosome-free fetal calf serum, aliquoted, and stored at −80°C until use. Purified viral stock titres were established using the Lentivirus-Associated p24 ELISA Kit (Cell Biolabs). Luciferase assays were performed with stock virus infected TZM-bl cells to determine virus replication and titres using a luciferase reporter gene assay system with extended-glow light emission kinetics (Steady Glo Luciferase Assay kit; Promega). LP-BM5 murine leukemia retrovirus (MLV) was prepared from the supernatant of a 7 day culture of the G6 clone of chronically infected SC-1 cells as previously described [33,54]. The properties and propagation of HSV-1 and HSV-2 have been previously described [55,56].

### Cells

The following cell lines were obtained through the NIH AIDS Reagent Program: PM1, TZM-bl, Sup-T1, Rev-CEM, H9, and U1/HIV-1. The U1/HIV-1 is a chronically infected cell line derived from limiting dilution cloning of U937 cells surviving acute infection with HIV-1_LAI/IIIB_. The Jurkat T lymphocyte cell line and the HEK 293 human embryonic kidney cell line were obtained from the American Tissue Culture Collection (ATCC). PM1, Sup-T1, Rev-CEM, and Jurkat cells were maintained in complete RPMI (cRPMI) 1640 (Gibco-BRL/Life Technologies) supplemented with 10% exosome depleted FCS (HyClone), 100 U/ml penicillin, 100 μg/ml streptomycin, sodium pyruvate and 0.3 mg/ml l-glutamine (Invitrogen, Molecular Probes). TZM-bl and HEK 293 cells were maintained in complete DMEM (cDMEM, Gibco-BRL/Life Technologies) supplemented with 10% exosome free FCS, 100 U/ml penicillin, 100 μg/ml streptomycin, sodium pyruvate and 0.3 mg/ml l-glutamine. Peripheral blood mononuclear cells (PBMCs) from several healthy anonymous blood donors were isolated from whole human blood using Ficoll-Hypaque density centrifugation according to the manufacturer’s instruction (Sigma-Aldrich). Monocytes were depleted from the PBMCs by adherence on gelatin-coated flasks as previously described [57]. The remaining peripheral blood leukocytes (PBLs) were propagated in cRPMI 1640 stimulated for 48 h with 5 μg/ml phytohemagglutinin (PHA, Roche Applied Science). Stimulated PBLs were washed with cRPMI and resuspended in fresh medium with interleukin-2 (IL-2; 10 U/ml). After 48 h, cells were used in experiments. SC1-G6 cells chronically infected with LP-BM5 (containing ecotropic, mink cell focus-forming and defective viruses) were obtained through the AIDS Research and Reference Reagent Program, and maintained in cDMEM. HEp-2 cells and Vero African green monkey kidney cells were from ATCC and were maintained in cDMEM. All cells were grown at 37°C with 5% CO_2._ Cell viability was assessed by CellTiter-Glo® luminescent cell viability assay (Promega Corporation).

### Electron microscopy

Transmission electron microscopy was done as previously described [58,59]. Exosomes were fixed in 2% paraformaldehyde and loaded onto formvar carbon coated grids. Exosomes adhered to carbon grids were PBS washed and immuno-stained with anti-CD63 antibody (BD Biosciences) or anti-Calnexin (EMD Millipore) overnight at 4°C. Grids were washed and incubated with 6 nm gold conjugated secondary antibody (Sigma-Aldrich) for 1 h at room temperature. Exosomes were then fixed with 2.5% glutaraldehyde, washed, contrasted in 1% uranyl acetate and imaged with a JEOL JEM 1230 transmission electron microscope.

### FACS analysis

4-μm diameter aldehyde/sulphate latex beads (Invitrogen, Molecular Probes) were washed in MES buffer and incubated with anti MHC-II MAb (provided by Dr. Nicholas Zavazava of the University of Iowa) or mouse IgG1 (Sigma-Aldrich) at room temperature overnight with gentle agitation. Exosomes (100 μg) were incubated at 4°C overnight with gentle agitation with 2 × 10^5^ anti MHC-II or mouse IgG1 coated latex beads. The reaction was stopped by 30 minute incubation with 200 mM glycine followed by three washes in FACS wash buffer (PBS with 1% exosome depleted FCS). Exosomes coated beads were then incubated with anti-CD63 conjugated to PE (Biolegend), anti-CD81 conjugated to FITC (Biolegend) or isotype control antibody for 1 h at room temperature and washed three times in FACS buffer. The resulting immunofluorescence was analysed by use of a FACSAria flow cytometer (Becton Dickinson) and Flowjo analysis software (TreeStar).

### Western blot analysis

Western blot was performed as previously described [42,60,61]. Briefly, for detection of CD63, calnexin and GAPDH, exosome preparations and spermatozoal extracts were lysed, resolved on a 12% SDS-PAGE, and stained with anti-CD63 (BD Biosciences), anti-GAPDH, or anti-Calnexin (EMD Millipore). The species-appropriate IRDye secondary antibodies were used followed by detection with the Odyssey Infrared Imaging System (LI-COR Biosciences). For detection of HIV-1 p24 and RT, monoclonal antibody to HIV-1 p24 (AG3.0) and anti-RT antibody (cat#6195) all obtained from NIH AIDS Reagent Program were used to probe immunoblot, followed by detection with the species-appropriate IRDye secondary antibodies, and imaged with Odyssey Infrared Imaging System.

### Real-time quantitative PCR

Quantitation of newly synthesized HIV-1 DNA was performed by Real-time quantitative PCR [40,43,60,62,63] using the 7500 fast real time PCR System (Applied Biosciences). Total DNA was extracted using the Wizard SV Genomic DNA purification kit (Promega). DNA concentration was determined spectrophotometrically and predetermined DNA was used to quantify viral cDNA intermediates using primers that amplify HIV-1 Gag [64]. Integration of HIV-1 proviral DNA was examined by nested PCR using genomic DNA isolated as described above. The Alu sense and HIV-1 Gag antisense primers were used for the first PCR and a predetermined amount of this PCR product was then used as template for nested PCR to detect HIV-1 LTR as previously described [65,66]. Exosome and spermatozoal RNA were isolated with Trizol RNA Reagent (AMRESCO) and treated with DNase (Qiagen). A portion of RNA was analyzed for RNA content and quality using Agilent Bioanalyzer instrument (Agilent). Another portion of RNA was subjected to cDNA synthesis (ABI) as previously described [33,58,60,62]. RNA concentration and purity were measured spectrophotometrically at 260/280 nm. Using synthesized cDNA, sequence-specific primers were used to amplify human CD9, CD63 and GAPDH while TaqMan primer/probes were used to detect APOBEC3 family members and BST-2/Tetherin as previously described [58,60,63]. PCR amplicons were visualized with ethidium bromide (Invitrogen, Molecular Probes) on 2% agarose gels in TAE buffer (Tris/EDTA/glacial acetic acid). LP-BM5 viral RNA and proviral DNA were determined as previously described [33,54]. Briefly, total RNA and DNA were isolated using a ZR-Duet™ DNA/RNA Mini Prep Kit (Zymo Research). Isolated RNA was exposed to DNAse and subjected to cDNA synthesis (ABI) as previously described [58-60,62,63]. Nucleic acid concentration and purity were measured spectrophotometrically at 260/280 nm. DNA and cDNA were amplified with primers and probe specific to BM5eco gag [33,54] and GAPDH using ABI 7500 FAST thermal cycler (ABI).

### *In vitro* exosome uptake

Exosomes were labeled in ultracentrifuge tubes at room temperature using the PKH67Green fluorescent kit (Sigma-Aldrich) according to the manufacturer’s instructions. Briefly, 100 μg of semen exosome (SE) or blood serum exosome (BE) in PBS was resuspended in 1 ml of diluent C, mixed with freshly prepared PKH67 in diluent C at a final concentration of 5 × 10-6 M and incubated for 3 min. Labeling was stopped by addition of an equal volume of exosome depleted FCS for 1 minute, followed by ultracentrifugation for 30 min at 100,000 × *g*. After two additional washes in PBS containing 10% exosome depleted FCS, the exosomes were resuspended in 100 μl of PBS. TZM-bL cells exposed to 100 μg/ml PKH67Green labelled SE or BE for 3 h and 24 h were washed thrice in PBS and collected using trypsin dissociation reagent (Gibco). Cells were fixed for 15 min on ice with 2% paraformaldehyde and fluorescence was analysed by use of a FACSCalibur flow cytometer (BD) to detect the PKH67Green transferred from exosomes to TZM-bl cells during fusion and uptake. Cellular frequency and fluorescence intensity were determined by Flowjo analysis software (TreeStar).

### HIV-1 infection

Semen is the vector for sexual transmission of HIV-1, and in an infected male, HIV-1 is expected to encounter SE in the male genital tract (MGT). Therefore, SE or BE (100 μg/mL) and HIV-1 (50 ng p24 antigen) were co-incubated for 1 h at 37°C prior to addition to cell cultures to mimic physiologic conditions in the MGT unless otherwise stated. TZM-bl cells are a genetically engineered HeLa cell line that expresses CD4, CXCR4 and CCR5 and contains HIV-1 Tat-inducible luciferase and β–Gal reporter genes. The level of HIV-1 infection was determined 24 h post viral challenge by resuspending TZM-bl cells in Steady-Glo® luciferase substrate (Promega Corporation) and measuring luminescence emission using a luminometer. Infectivity readout was also assessed using the Lentivirus-Associated p24 ELISA Kit (Cell Biolabs), EnzChek® Reverse Transcriptase Assay Kit (Life Technologies Corporation), and RT-qPCR. Where indicated, HIV-1 target cells were pretreated with or without 10 nM AZT for 2 h at 37°C prior to infection; and AZT was maintained for the duration of viral challenge. Viral input was washed from cells thrice with PBS at 3 h post infection. For assessment of viral entry at 3 h post exposure TZM-bL cell monolayers were washed thrice in PBS and trypsinized (0.05%, Gibco) to efficiently dissociate virus bound to the extracellular membrane [34] and to dissociate the cells from the plate followed by three PBS washing steps in a Eppendorf tubes at 300 × g for 5 m to eliminate input virus that was not internalized. Cells were then lysed with exosome free complete medium containing 2% Triton-X 100 (Sigma-Aldrich) and pelleted by centrifugation at 10,000 × g for 10 m. Supernatant was collected and used to assay HIV internalization by p24 ELISA and RT activity.

### LP-BM5 infection

Splenocytes obtained from healthy C57BL/6 mice were plated at 1 × 10^5^ in triplicates and infected with LP-BM5 that had been pre-incubated with PBS or SE (100 μg/mL) for 1 h at 37°C. Input virus was washed from cells thrice with PBS at 3 h post infection. Ninety-six hours post viral challenge, total DNA was extracted from cells and used to evaluate viral load by quantitative PCR detection of LP-BM5 proviral DNA.

### HSV-1 and HSV-2 plaque reduction and single step growth assays

For plaque reduction assay, 200,000 PFU of either HSV-1(F) or HSV-2(G) [55] were incubated in 250 μl infection medium (DMEM containing 1% heat inactivated calf serum) with no exosomes (PBS vehicle), 100 μg/mL of BE or 100 μg/mL of SE at 37°C for 1 hour. The volume of each reaction was brought to 1 ml with infection medium, and then serial ten-fold dilutions were prepared in infection medium. Inocula containing 1 ml of the 10–1, 10–2, and 10–3 dilutions were plated onto confluent six-well cultures of HEp-2 cells and incubated for 90 min at 37°C, after which the inocula were replaced with infection medium containing 1% methylcellulose. Plaques were allowed to develop for two days at 37°C, and were then fixed, immuno-stained and counted as previously described [56].

For the single-step growth assay, HEp-2 cells were pre-incubated at 37°C for 1 hour with no exosomes (PBS vehicle), 100 μg/mL of BE or 100 μg/mL of SE. HSV-1 or HSV-2 (MOI = 5) was added to the cells and incubated for 1 hour at 37°C. Residual virus was removed by washing with citrate buffer at pH =3.0 and cells were incubated for 18 hours to allow virus replication. Stocks were prepared from each of the infected cultures by freeze-thaw and then each stock was titered on Vero cells.

### Statistical analysis

Statistical analysis of significant differences was tested using paired, two-tailed Student’s *t* test. A *p-*value of ≤0.05 was regarded as statistically significant. Error bars represent standard deviations (SD).

## Abbreviations

HIV-1: Human immunodeficiency virus type 1
SE: Semen-derived exosomes
BE: Blood-derived exosomes
MGT: Male genital tract
FRT: Female reproductive tract
TEM: Transmission electron microscopy
FACS: Fluorescence-activated cell sorting
HRFs: Host restriction factors
RT: Reverse transcriptase
A3: APOBEC3, Apolipoprotein B mRNA-editing, enzyme-catalytic, polypeptide-like 3
PBL: Peripheral blood leukocytes
DNA: Deoxyribonucleic acid
RNA: Ribonucleic acid
AZT: Azidothymidine/zidovudine/Retrovir
T/F: Transmitted/founder
BST-2: Bone marrow stromal cell antigen 2

## References

[1] Simpson RJ, Jensen SS, Lim JW (2008). Proteomic profiling of exosomes: current perspectives. Proteomics.

[2] Schorey JS, Bhatnagar S (2008). Exosome function: from tumor immunology to pathogen biology. Traffic.

[3] Alvarez-Erviti L, Seow Y, Yin H, Betts C, Lakhal S, Wood MJ (2011). Delivery of siRNA to the mouse brain by systemic injection of targeted exosomes. Nat Biotechnol.

[4] Caby MP, Lankar D, Vincendeau-Scherrer C, Raposo G, Bonnerot C (2005). Exosomal-like vesicles are present in human blood plasma. Int Immunol.

[5] Pisitkun T, Shen RF, Knepper MA (2004). Identification and proteomic profiling of exosomes in human urine. Proc Natl Acad Sci U S A.

[6] Palanisamy V, Sharma S, Deshpande A, Zhou H, Gimzewski J, Wong DT (2010). Nanostructural and transcriptomic analyses of human saliva derived exosomes. PLoS One.

[7] Admyre C, Johansson SM, Qazi KR, Filen JJ, Lahesmaa R, Norman M, Neve EP, Scheynius A, Gabrielsson S (2007). Exosomes with immune modulatory features are present in human breast milk. J Immunol.

[8] Admyre C, Grunewald J, Thyberg J, Gripenback S, Tornling G, Eklund A, Scheynius A, Gabrielsson S (2003). Exosomes with major histocompatibility complex class II and co-stimulatory molecules are present in human BAL fluid. Eur Respir J.

[9] Poliakov A, Spilman M, Dokland T, Amling CL, Mobley JA (2009). Structural heterogeneity and protein composition of exosome-like vesicles (prostasomes) in human semen. Prostate.

[10] Ronquist G, Brody I (1985). The prostasome: its secretion and function in man. Biochim Biophys Acta.

[11] Conde-Vancells J, Rodriguez-Suarez E, Embade N, Gil D, Matthiesen R, Valle M, Elortza F, Lu SC, Mato JM, Falcon-Perez JM (2008). Characterization and comprehensive proteome profiling of exosomes secreted by hepatocytes. J Proteome Res.

[12] Subra C, Grand D, Laulagnier K, Stella A, Lambeau G, Paillasse M, De Medina P, Monsarrat B, Perret B, Silvente-Poirot S, Poirot M, Record M (2010). Exosomes account for vesicle-mediated transcellular transport of activatable phospholipases and prostaglandins. J Lipid Res.

[13] Vojtech L, Woo S, Hughes S, Levy C, Ballweber L, Sauteraud RP, Strobl J, Westerberg K, Gottardo R, Tewari M, Hladik F (2014). Exosomes in human semen carry a distinctive repertoire of small non-coding RNAs with potential regulatory functions. Nucleic Acids Res.

[14] Mittelbrunn M, Gutierrez-Vazquez C, Villarroya-Beltri C, Gonzalez S, Sanchez-Cabo F, Gonzalez MA, Bernad A, Sanchez-Madrid F (2011). Unidirectional transfer of microRNA-loaded exosomes from T cells to antigen-presenting cells. Nat Commun.

[15] Skog J, Wurdinger T, van Rijn S, Meijer DH, Gainche L, Sena-Esteves M, Curry WT, Carter BS, Krichevsky AM, Breakefield XO (2008). Glioblastoma microvesicles transport RNA and proteins that promote tumour growth and provide diagnostic biomarkers. Nat Cell Biol.

[16] Zomer A, Vendrig T, Hopmans ES, van Eijndhoven M, Middeldorp JM, Pegtel DM (2010). Exosomes: fit to deliver small RNA. Commun Integr Biol.

[17] Sun D, Zhuang X, Xiang X, Liu Y, Zhang S, Liu C, Barnes S, Grizzle W, Miller D, Zhang HG (2010). A novel nanoparticle drug delivery system: the anti-inflammatory activity of curcumin is enhanced when encapsulated in exosomes. Mol Ther.

[18] Khatua AK, Taylor HE, Hildreth JE, Popik W (2009). Exosomes packaging APOBEC3G confer human immunodeficiency virus resistance to recipient cells. J Virol.

[19] Li J, Liu K, Liu Y, Xu Y, Zhang F, Yang H, Liu J, Pan T, Chen J, Wu M, Zhou X, Yuan Z (2013). Exosomes mediate the cell-to-cell transmission of IFN-alpha-induced antiviral activity. Nat Immunol.

[20] Kelly RW, Critchley HO (1997). Immunomodulation by human seminal plasma: a benefit for spermatozoon and pathogen?. Hum Reprod.

[21] Royce RA, Sena A, Cates W, Cohen MS (1997). Sexual transmission of HIV. N Engl J Med.

[22] Varghese B, Maher JE, Peterman TA, Branson BM, Steketee RW (2002). Reducing the risk of sexual HIV transmission: quantifying the per-act risk for HIV on the basis of choice of partner, sex act, and condom use. Sex Transm Dis.

[23] O’Brien TR, Busch MP, Donegan E, Ward JW, Wong L, Samson SM, Perkins HA, Altman R, Stoneburner RL, Holmberg SD (1994). Heterosexual transmission of human immunodeficiency virus type 1 from transfusion recipients to their sex partners. J Acquir Immune Defic Syndr.

[24] Martellini JA, Cole AL, Svoboda P, Stuchlik O, Chen LM, Chai KX, Gangrade BK, Sorensen OE, Pohl J, Cole AM (2011). HIV-1 enhancing effect of prostatic acid phosphatase peptides is reduced in human seminal plasma. PLoS One.

[25] Martellini JA, Cole AL, Venkataraman N, Quinn GA, Svoboda P, Gangrade BK, Pohl J, Sorensen OE, Cole AM (2009). Cationic polypeptides contribute to the anti-HIV-1 activity of human seminal plasma. FASEB J.

[26] Munch J, Rucker E, Standker L, Adermann K, Goffinet C, Schindler M, Wildum S, Chinnadurai R, Rajan D, Specht A, Giménez-Gallego G, Sánchez PC, Fowler DM, Koulov A, Kelly JW, Mothes W, Grivel JC, Margolis L, Keppler OT, Forssmann WG, Kirchhoff F (2007). Semen-derived amyloid fibrils drastically enhance HIV infection. Cell.

[27] Roan NR, Liu H, Usmani SM, Neidleman J, Muller JA, Avila-Herrera A, Gawanbacht A, Zirafi O, Chu S, Dong M, Kumar ST, Smith JF, Pollard KS, Fändrich M, Kirchhoff F, Münch J, Witkowska HE, Greene WC (2014). Liquefaction of semen generates and later degrades a conserved semenogelin peptide that enhances HIV infection. J Virol.

[28] Keller S, Sanderson MP, Stoeck A, Altevogt P (2006). Exosomes: from biogenesis and secretion to biological function. Immunol Lett.

[29] Kesimer M, Scull M, Brighton B, DeMaria G, Burns K, O’Neal W, Pickles RJ, Sheehan JK (2009). Characterization of exosome-like vesicles released from human tracheobronchial ciliated epithelium: a possible role in innate defense. FASEB J.

[30] Keller S, Ridinger J, Rupp AK, Janssen JW, Altevogt P (2011). Body fluid derived exosomes as a novel template for clinical diagnostics. J Transl Med.

[31] Kogure T, Lin WL, Yan IK, Braconi C, Patel T (2011). Intercellular nanovesicle-mediated microRNA transfer: a mechanism of environmental modulation of hepatocellular cancer cell growth. Hepatology.

[32] Naslund TI, Paquin-Proulx D, Paredes PT, Vallhov H, Sandberg JK, Gabrielsson S (2014). Exosomes from breast milk inhibit HIV-1 infection of dendritic cells and subsequent viral transfer to CD4+ T cells. AIDS.

[33] Jones PH, Mehta HV, Okeoma CM (2012). A novel role for APOBEC3: Susceptibility to sexual transmission of murine acquired immunodeficiency virus (mAIDS) is aggravated in APOBEC3 deficient mice. Retrovirology.

[34] Tang SB, Levy JA (1991). Inactivation of HIV-1 by trypsin and its use in demonstrating specific virus infection of cells. J Virol Methods.

[35] Marozsan AJ, Fraundorf E, Abraha A, Baird H, Moore D, Troyer R, Nankja I, Arts EJ (2004). Relationships between infectious titer, capsid protein levels, and reverse transcriptase activities of diverse human immunodeficiency virus type 1 isolates. J Virol.

[36] Ajamian L, Abrahamyan L, Milev M, Ivanov PV, Kulozik AE, Gehring NH, Mouland AJ (2008). Unexpected roles for UPF1 in HIV-1 RNA metabolism and translation. RNA.

[37] Butsch M, Boris-Lawrie K (2000). Translation is not required To generate virion precursor RNA in human immunodeficiency virus type 1-infected T cells. J Virol.

[38] Tachedjian G, Radzio J, Sluis-Cremer N (2005). Relationship between enzyme activity and dimeric structure of recombinant HIV-1 reverse transcriptase. Proteins.

[39] Tachedjian G, Aronson HE, de los Santos M, Seehra J, McCoy JM, Goff SP (2003). Role of residues in the tryptophan repeat motif for HIV-1 reverse transcriptase dimerization. J Mol Biol.

[40] Okeoma CM, Lovsin N, Peterlin BM, Ross SR (2007). APOBEC3 inhibits mouse mammary tumour virus replication in vivo. Nature.

[41] Low A, Okeoma CM, Lovsin N, de las Heras M, Taylor TH, Peterlin BM, Ross SR, Fan H (2009). Enhanced replication and pathogenesis of Moloney murine leukemia virus in mice defective in the murine APOBEC3 gene. Virology.

[42] Okeoma CM, Huegel AL, Lingappa J, Feldman MD, Ross SR (2010). APOBEC3 proteins expressed in mammary epithelial cells are packaged into retroviruses and can restrict transmission of milk-borne virions. Cell Host Microbe.

[43] Okeoma CM, Low A, Bailis W, Fan HY, Peterlin BM, Ross SR (2009). Induction of APOBEC3 in vivo causes increased restriction of retrovirus infection. J Virol.

[44] Okeoma CM, Petersen J, Ross SR (2009). Expression of murine APOBEC3 alleles in different mouse strains and their effect on mouse mammary tumor virus infection. J Virol.

[45] Iwatani Y, Chan DS, Wang F, Maynard KS, Sugiura W, Gronenborn AM, Rouzina I, Williams MC, Musier-Forsyth K, Levin JG (2007). Deaminase-independent inhibition of HIV-1 reverse transcription by APOBEC3G. Nucleic Acids Res.

[46] Mbisa JL, Barr R, Thomas JA, Vandegraaff N, Dorweiler IJ, Svarovskaia ES, Brown WL, Mansky LM, Gorelick RJ, Harris RS, Engelman A, Pathak VK (2007). Human immunodeficiency virus type 1 cDNAs produced in the presence of APOBEC3G exhibit defects in plus-strand DNA transfer and integration. J Virol.

[47] Wang X, Ao Z, Chen L, Kobinger G, Peng J, Yao X (2012). The cellular antiviral protein APOBEC3G interacts with HIV-1 reverse transcriptase and inhibits its function during viral replication. J Virol.

[48] de Hoog VC, Timmers L, Schoneveld AH, Wang JW, van de Weg SM, Sze SK, van Keulen JK, Hoes AW, den Ruijter HM, de Kleijn DP, Mosterd A (2013). Serum extracellular vesicle protein levels are associated with acute coronary syndrome. Eur Heart J Acute Cardiovasc Care.

[49] Quackenbush JF, Cassidy PB, Pfeffer LM, Boucher KM, Hawkes JE, Pfeffer SR, Kopelovich L, Leachman SA (2014). Isolation of circulating MicroRNAs from Microvesicles found in human plasma. Methods Mol Biol.

[50] Rekker K, Saare M, Roost AM, Kubo AL, Zarovni N, Chiesi A, Salumets A, Peters M (2013). Comparison of serum exosome isolation methods for microRNA profiling. Clin Biochem.

[51] Booth AM, Fang Y, Fallon JK, Yang JM, Hildreth JE, Gould SJ (2006). Exosomes and HIV Gag bud from endosome-like domains of the T cell plasma membrane. J Cell Biol.

[52] Liao Z, Graham DR, Hildreth JE (2003). Lipid rafts and HIV pathogenesis: virion-associated cholesterol is required for fusion and infection of susceptible cells. AIDS Res Hum Retroviruses.

[53] Ochsenbauer C, Edmonds TG, Ding H, Keele BF, Decker J, Salazar MG, Salazar-Gonzalez JF, Shattock R, Haynes BF, Shaw GM, Hahn BH, Kappes JC (2012). Generation of transmitted/founder HIV-1 infectious molecular clones and characterization of their replication capacity in CD4 T lymphocytes and monocyte-derived macrophages. J Virol.

[54] Cook WJ, Green KA, Obar JJ, Green WR (2003). Quantitative analysis of LP-BM5 murine leukemia retrovirus RNA using real-time RT-PCR. J Virol Methods.

[55] Ejercito PM, Kieff ED, Roizman B (1968). Characterization of herpes simplex virus strains differing in their effects on social behaviour of infected cells. J Gen Virol.

[56] Roller RJ, Haugo AC, Yang K, Baines JD (2014). The herpes simplex virus 1 UL51 gene product has cell type-specific functions in cell-to-cell spread. J Virol.

[57] Maury W (1994). Monocyte maturation controls expression of equine infectious anemia virus. J Virol.

[58] Jones PH, Maric M, Madison MN, Maury W, Roller RJ, Okeoma CM (2013). BST-2/tetherin-mediated restriction of chikungunya (CHIKV) VLP budding is counteracted by CHIKV non-structural protein 1 (nsP1). Virology.

[59] Jones PH, Mehta HV, Maric M, Roller RJ, Okeoma CM (2012). Bone marrow stromal cell antigen 2 (BST-2) restricts mouse mammary tumor virus (MMTV) replication in vivo. Retrovirology.

[60] Mehta HV, Jones PH, Weiss JP, Okeoma CM (2012). IFN-alpha and lipopolysaccharide upregulate APOBEC3 mRNA through different signaling pathways. J Immunol.

[61] Okeoma CM, Shen M, Ross SR (2008). A novel block to mouse mammary tumor virus infection of lymphocytes in B10.BR mice. J Virol.

[62] Jones PH, Mahauad-Fernandez WD, Madison MN, Okeoma CM (2013). BST-2/tetherin is overexpressed in mammary gland and tumor tissues in MMTV-induced mammary cancer. Virology.

[63] Jones PH, Okeoma CM (2013). Phosphatidylinositol 3-kinase is involved in Toll-like receptor 4-mediated BST-2/tetherin regulation. Cell Signal.

[64] Li XY, Guo F, Zhang L, Kleiman L, Cen S (2007). APOBEC3G inhibits DNA strand transfer during HIV-1 reverse transcription. J Biol Chem.

[65] Masuda T, Planelles V, Krogstad P, Chen IS (1995). Genetic analysis of human immunodeficiency virus type 1 integrase and the U3 att site: unusual phenotype of mutants in the zinc finger-like domain. J Virol.

[66] Zack JA, Arrigo SJ, Weitsman SR, Go AS, Haislip A, Chen IS (1990). HIV-1 entry into quiescent primary lymphocytes: molecular analysis reveals a labile, latent viral structure. Cell.

